# Chiral Vibrational Structures of Proteins at Interfaces Probed by Sum Frequency Generation Spectroscopy

**DOI:** 10.3390/ijms12129404

**Published:** 2011-12-16

**Authors:** Li Fu, Zhuguang Wang, Elsa C.Y. Yan

**Affiliations:** Department of Chemistry, Yale University, 225 Prospect Street, New Haven, CT, 06520, USA; E-Mails: li.fu@yale.edu (L.F.); zhuguang.wang@yale.edu (Z.W.)

**Keywords:** chirality, chiral sum frequency generation spectroscopy, amide I, N-H stretch, protein secondary structures, chiral vibrational structures of proteins, interfaces

## Abstract

We review the recent development of chiral sum frequency generation (SFG) spectroscopy and its applications to study chiral vibrational structures at interfaces. This review summarizes observations of chiral SFG signals from various molecular systems and describes the molecular origins of chiral SFG response. It focuses on the chiral vibrational structures of proteins and presents the chiral SFG spectra of proteins at interfaces in the C-H stretch, amide I, and N-H stretch regions. In particular, a combination of chiral amide I and N-H stretches of the peptide backbone provides highly characteristic vibrational signatures, unique to various secondary structures, which demonstrate the capacity of chiral SFG spectroscopy to distinguish protein secondary structures at interfaces. On the basis of these recent developments, we further discuss the advantages of chiral SFG spectroscopy and its potential application in various fields of science and technology. We conclude that chiral SFG spectroscopy can be a new approach to probe chiral vibrational structures of protein at interfaces, providing structural and dynamic information to study *in situ* and in real time protein structures and dynamics at interfaces.

## 1. Introduction

Chirality plays an important role in modern chemistry, biology, and medicine. Molecules in biological systems, such as sugars, nucleic acids, and amino acids have inherent chiral centers. These molecules polymerize into polysaccharides, DNAs and RNAs, and proteins, which further adopt chiral secondary and tertiary structures. Among these biopolymers, proteins fold into highly specific structures according to their amino acid sequence. These structures allow them to function as molecular machinery to perform various biological functions in almost every aspect of life. The chiral secondary and tertiary structures of proteins have long been of interest to researchers. Optical methods have been developed to characterize protein chiral structures, yielding molecular information to solve fundamental and engineering problems in biomedical and material sciences.

In the past century, much progress has been made in the development of chiroptical techniques, allowing us to study molecular chirality using circular dichroism (CD) [1–4], optical rotation dispersion (ORD) [4–6], Raman optical activity (ROA) [7–9], *etc*. As the diversity of techniques grows, more advanced optical methods, such as time-resolved CD [10–13], vibrational CD [14–16], and twophoton absorption CD [17–19], have become available to achieve higher time resolution, sensitivity, and structural selectivity. However, it remains difficult to characterize chiral properties of biomolecules *in situ* and in real time at interfaces. This is mainly due to the lack of optical methods that have both sensitivity to chirality and selectivity to interfaces.

Recently, nonlinear chiroptical phenomena have introduced a new means to characterize chiral surfaces. In the 1990s, the Hicks group and the Persoons group observed chiral effects using second harmonic generation (SHG) spectroscopy [20–25]. Since then, the importance of chiroptical response in nonlinear spectroscopy has been increasingly recognized due to its high selectivity to interfaces and sensitivity to molecular chirality. Representative nonlinear chiroptical techniques include SHG linear dichroism (SHG-LD) [21,26,27] and SHG circular dichroism (SHG-CD) [27–29], nonlinear optical activity (NOA) [30,31], and chiral sum frequency generation (chiral SFG) [32–35]. These studies have revealed that many biomolecules exhibit nonlinear chiroptical activity, leading to various applications in structural analysis [35–38], material sciences [39,40], and imaging [41–43]. These applications have demonstrated that chiral effects in nonlinear spectroscopy are selective and sensitive probes for biological systems, presenting exciting opportunities for structural analysis of chiral surfaces using nonlinear spectroscopy.

In the past decade, chiral SFG spectroscopy has been developed to probe chiral vibrational structures of biomolecules. In this review, we summarize the recent development of chiral sum frequency generation (SFG) spectroscopy and its applications to study chiral vibrational structures at interfaces. We first discuss general theory of chiral SFG and the molecular origins of chiral signals from proteins. Next, we describe the instruments and experimental approaches for using chiral SFG to probe proteins at interfaces. Subsequently, we summarize the up-to-date results of chiral vibrational studies of proteins at interfaces. These spectra include chiral C-H, amide I, and N-H stretches of various model proteins and peptides at interfaces. In particular, the amide I and N-H stretch spectra of peptide backbone show highly characteristic vibrational signatures unique to specific secondary structures. These results reveal the capacity of chiral SFG for characterizing protein secondary structures at interfaces. This capacity is further demonstrated by a recent study using chiral SFG to probe real-time kinetics of folding and aggregation of amyloid proteins at interfaces. These developments support that chiral SFG spectroscopy can be used to distinguish between secondary structures at interfaces, similar to the use of CD spectroscopy for characterizing protein secondary structures in bulk solution.

## 2. General Principles of Chiral Sum Frequency Generation Spectroscopy

### 2.1. SFG Method

Rigorous treatment of SFG theory can be found in excellent reviews [44–46] and books [47,48]. Here, we focus on describing the theory to illustrate how SFG can be used as a surface-specific vibrational spectroscopy. SFG uses two pulsed laser beams, one at infrared (IR) frequency *ω_IR_* and the other at visible frequency *ω_VIS_*. When these two beams spatially and temporally overlap at surfaces, a second-order polarization, *P^(2)^*, at the sum frequency (*ω_IR_* *+ ω_Vis_*) can be induced to generate an SFG signal. The intensity of the SFG signal is related to the surface second order susceptibility, *χ*^(2)^, and the electric fields of the IR and visible beams as below,

(1) $$I_{SFG}\propto {\left|\chi^{\left(2\right)}E_{IR}E_{VIS}\right|}^{2}$$

**Figure 1:**
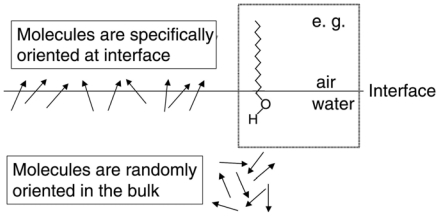
The noncentrosymmetric property of interfaces: Hydrophobic and hydrophilic interactions orient a long chain alcohol molecule at the air-water interface, and the arrows represent the transition dipole of molecules under study.

Under the dipole approximation, *χ^(2)^* is nonzero only if the medium lacks centrosymmetry (Figure 1). This is the case for interfaces because molecules align due to asymmetric physical and chemical properties of two media. The second-order susceptibility of an interface consists of a non-resonant term, *χ_NR_*^(2)^, and a sum of vibrational resonant terms, *χ_q_*^(2)^, which is considered to be in a Lorentzian lineshape as shown below,

(2) $$\chi^{\left(2\right)}=\chi_{NR}^{\left(2\right)}+\sum_{q}\chi_{q}^{\left(2\right)}=\chi_{NR}^{\left(2\right)}+\sum_{q}\frac{A_{q}}{\omega_{IR}-\omega_{q}+i\Gamma_{q}}$$

where *A_q_* is the amplitude, *Γ_q_* is the damping coefficient, and *ω_q_* is the vibrational frequency of the *q^th^* vibrational mode, and *ω_IR_* is the incident IR frequency. The SFG signal is enhanced when *ω_IR_* is in resonance with *ω_q_*. Therefore, SFG is a second-order surface-specific vibrational spectroscopy.

### 2.2. Chiral SFG

Furthermore, one can use SFG to selectively probe the chirality of interfaces. The selectivity of chirality comes from the coherent nature of the SFG signals, which is directly related to a third-rank tensor of 2^nd^-order nonlinear optical susceptibility, *χ*^(2)^, as shown in Equation 1. SFG experiments are often performed using linearly *s*- or *p*-polarized IR and visible beams, and measuring *s-* or *p-*polarized SFG signals. Hence, there are eight possible combinations for the polarization settings: *ssp* (*s*-polarized SFG, *s*-polarized visible, and *p*-polarized IR), *sps*, *pss*, *ppp*, *sss, psp*, *spp*, and *pps*. For a particular polarization setting, the SFG intensity (*I_SFG_*) can be expressed by an effective second-order susceptibility, *χ_eff_*^(2)^, e.g., *χ_ssp_*^(2)^, *χ_ppp_*^(2)^, *χ_psp_*^(2)^, *etc.*

(3) $$I_{SFG}\propto {\left|\chi_{eff}^{\left(2\right)}\right|}^{2}$$

The effective second-order susceptibility, *χ_eff_*^(2)^, is a linear combination of the *χ^(2)^* tensor elements, *χ_IJK_*^(2)^, where *I*, *J*, *K* are laboratory coordinates (*x*, *y*, *z*). For an isotropic achiral surface with the *C_∞V_* symmetry, there are only seven nonzero *χ_IJK_*^(2)^ elements: *χ_xxz_*^(2)^ = *χ_yyz_*^(2)^, *χ_xzx_*^(2)^ = *χ_yzy_*^(2)^, *χ_zxx_*^(2)^ = *χ_zyy_*^(2),^ and *χ_zzz_*^(2)^. For a chiral surface with the *C_∞_* symmetry, there are six additional nonzero elements: *χ_xyz_*^(2)^, *χ_yxz_*^(2),^ *χ_zxy_*^(2)^, *χ_zyx_*^(2),^ *χ_xzy_*^(2)^ and *χ_yzx_*^(2)^. These orthogonal elements, *χ_IJK_*^(2)^ (*I* ≠ *J* ≠ *K*), are characteristic of chiral surfaces. These chiral and achiral elements can be used to express eight effective *χ_eff_*^(2)^, of which five are achiral as shown in Equation 4 and three are chiral as shown in Equation 5 [34,49]:

(4) $$\begin{array}{l} \chi_{ssp}^{\left(2\right)}=L_{yy}(\omega_{SFG})L_{yy}(\omega_{VIS})L_{zz}(\omega_{IR})\text{sin}\;\alpha_{IR}\;\chi_{yyz}^{\left(2\right)}\\ \chi_{sps}^{\left(2\right)}=L_{yy}(\omega_{SFG})L_{zz}(\omega_{VIS})L_{yy}(\omega_{IR})\text{sin}\;\alpha_{VIS}\;\chi_{yzy}^{\left(2\right)}\\ \chi_{pss}^{\left(2\right)}=L_{zz}(\omega_{SFG})L_{yy}(\omega_{VIS})L_{yy}(\omega_{IR})\text{sin}\;\alpha_{SFG}\;\chi_{zyy}^{\left(2\right)}\\ \chi_{ppp}^{\left(2\right)}=-L_{xx}(\omega_{SFG})L_{xx}(\omega_{VIS})L_{zz}(\omega_{IR})\text{cos}\;\alpha_{SFG}\;\text{cos}\;\alpha_{VIS}\;\text{sin}\;\alpha_{IR}\;\chi_{xxz}^{\left(2\right)}\\ -L_{xx}(\omega_{SFG})L_{zz}(\omega_{VIS})L_{xx}(\omega_{IR})\text{cos}\;\alpha_{SFG}\;\text{sin}\;\alpha_{VIS}\;\text{cos}\;\alpha_{IR}\;\chi_{xzx}^{\left(2\right)}\\ +L_{zz}(\omega_{SFG})L_{xx}(\omega_{VIS})L_{xx}(\omega_{IR})\text{cos}\;\alpha_{SFG}\;\text{sin}\;\alpha_{VIS}\;\text{cos}\;\alpha_{IR}\;\chi_{zxx}^{\left(2\right)}\\ +L_{zz}(\omega_{SFG})L_{zz}(\omega_{VIS})L_{zz}(\omega_{IR})\text{sin}\;\alpha_{SFG}\;\text{sin}\;\alpha_{VIS}\;\text{sin}\;\alpha_{IR}\;\chi_{zzz}^{\left(2\right)} \end{array}$$

(5) $$\begin{array}{l} \chi_{ssp}^{\left(2\right)}=0\\ \chi_{psp}^{\left(2\right)}=L_{zz}(\omega_{SFG})L_{yy}(\omega_{VIS})L_{xx}(\omega_{IR})\text{sin}\;\alpha_{SFG}\;\text{cos}\;\alpha_{IR}\;\chi_{zyx}^{\left(2\right)}\\ -L_{xx}(\omega_{SFG})L_{yy}(\omega_{VIS})L_{zz}(\omega_{IR})\text{cos}\;\alpha_{SFG}\;\text{sin}\;\alpha_{IR}\;\chi_{xyz}^{\left(2\right)}\\ \chi_{spp}^{\left(2\right)}=L_{yy}(\omega_{SFG})L_{zz}(\omega_{VIS})L_{xx}(\omega_{IR})\text{sin}\;\alpha_{VIS}\;\text{cos}\;\alpha_{IR}\;\chi_{yzx}^{\left(2\right)}\\ -L_{yy}(\omega_{SFG})L_{xx}(\omega_{VIS})L_{zz}(\omega_{IR})\text{cos}\;\alpha_{SFG}\;\text{sin}\;\alpha_{VIS}\;\chi_{yxz}^{\left(2\right)}\\ \chi_{pps}^{\left(2\right)}=L_{zz}(\omega_{SFG})L_{xx}(\omega_{VIS})L_{yy}(\omega_{IR})\text{sin}\;\alpha_{SFG}\;\text{cos}\;\alpha_{VIS}\;\chi_{zxy}^{\left(2\right)}\\ -L_{xx}(\omega_{SFG})L_{zz}(\omega_{VIS})L_{yy}(\omega_{IR})\text{cos}\;\alpha_{SFG}\;\text{sin}\;\alpha_{VIS}\;\chi_{xzy}^{\left(2\right)} \end{array}$$

where *α_i_* is the incident or reflected angle of the *i*^th^ laser beam and *L(ω_i_)* is the Fresnel factor. Equation 5 shows three chiral effective susceptibilities, *χ_psp_*^(2)^, *χ_spp_*^(2)^, and *χ_pps_*^(2)^, which contain only the orthogonal chiral tensor elements *χ_IJK_*^(2)^ (*I* ≠ *J* ≠ *K, )*, including *χ_xyz_*^(2)^, *χ_yxz_*^(2),^ *χ_zxy_*^(2)^, *χ_zyx_*^(2),^ *χ_xzy_*^(2)^ and *χ_yzx_*^(2)^. Thus, surface chirality can be probed by using the *psp*, *spp*, and *pps* polarizations without interference from the background of achiral solute and solvent molecules at the interface.

**Figure 2:**
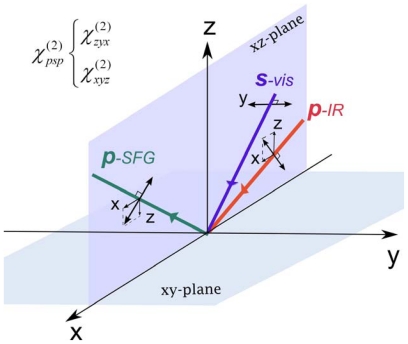
The *psp* polarization for chiral SFG measurements.

Among these polarization configurations, the *psp* polarization is commonly used to obtain chiral SFG spectra. As shown in Figure 2, when the *psp* polarization setting (*p*-polarized SFG, *s*-polarized visible, and *p*-polarized IR) is used, the *p*-polarized SFG and the *p*-polarized IR light have electric fields in the *x* and *z* directions such that the first (*I*) and third index (*K*) of *χ_IJK_*^(2)^ denoting the direction of the electric fields of the SFG signal and the IR beam, respectively, can only be either *z* or *x*. The *s*-polarized visible beam has an electric field only in the *y* direction such that the second index (*J*) of *χ_IJK_*^(2)^ denoting the direction of an electric field, can only be *y*. Thus, the *psp* polarization setting can be used to measure two chiral elements *χ_xyz_*^(2)^ and *χ_zyx_*^(2)^. A combination of Equations 3 and 5 describes how the experimental observable (*I_SFG_*) relates to the two chiral second-order susceptibility tensor elements *χ_xyz_*^(2)^ and *χ_zyx_*^(2)^.

### 2.3. Hyperpolarizability and Molecular Orientation

**Figure 3:**
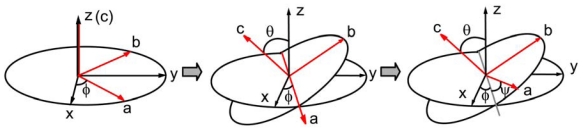
The Euler transformation from the molecular coordinates (*a*, *b*, *c*) to the laboratory coordinates (*x*, *y*, *z*).

The experimental observable *I_SFG_* can be further related to microscopic hyperpolarizability (*β*) of molecules at interfaces because macroscopic *χ_IJK_*^(2)^ elements are ensemble averages of microscopic *β* tensor elements, *β_ijk_*. The *χ_IJK_*^(2)^ elements can be expressed by *β_ijk_* using the Euler transformation as shown in Figure 3 and Equation 6, where *I*, *J*, *K* are the laboratory coordinates (*x*, *y*, *z*) and *i*, *j*, *k* are molecular coordinates (*a*, *b*, *c*):

(6) $$\chi_{IJK,q}^{\left(2\right)}=N_{s}\sum_{i,j,k}\langle R_{Ii}R_{Jj}R_{Kk}\rangle \beta_{ijk,q}$$

where *N_s_* is the number density of the interface moiety under study and *R_Ii_*, *R_Jj_*, and *R_Kk_* are elements of the rotational transformation matrix from the molecular coordinates to the laboratory coordinates (Figure 3). The microscopic *β* tensor determines the SFG response of a molecule, which is related to the electric polarizability (*α_ij_*) and electric dipole moment (*μ_k_*),

(7) $$\beta_{ijk,q}\propto \frac{\partial \alpha_{ij}}{\partial Q_{q}}\frac{\partial \mu_{k}}{\partial Q_{q}}$$

where *Q_q_* is the *q^th^* normal mode coordinate. Equation 7 shows that the vibrational mode that is SFG active must be both IR and Raman active.

The intensity of the chiral SFG signal measured using the *psp* polarization is related to *χ_psp_*^(2)^ according to Equation 3, which is further related to two chiral elements *χ_xyz_*^(2)^ and *χ_zyx_*^(2)^ according to Equation 5. The expression of these two chiral elements, as shown in Equations 8 and 9, can be obtained using Equation 6, which introduces the molecular orientation (*θ*, *ψ*) into the equation by averaging over the in-plane orientation angle (*Φ*) from *0* to *2π*[50].

(8) $$\begin{array}{l} \chi_{zyx}^{\left(2\right)}=-\chi_{zxy}^{\left(2\right)}=-\frac{1}{2}N_{S}\times \\ \left\{\begin{array}{l} \langle {\text{cos}}^{2}\;\theta \rangle (\beta_{cab}-\beta_{cba})\\ +\langle {\text{sin}}^{2}\;\theta \;{\text{sin}}^{2}\;\psi \rangle (\beta_{bca}-\beta_{bac})\\ +\langle {\text{sin}}^{2}\;\theta \;{\text{cos}}^{2}\;\psi \rangle (\beta_{abc}-\beta_{acb})\\ +\langle {\text{sin}}^{2}\;\theta \;\text{sin}\;\psi \;\text{cos}\;\psi \rangle (\beta_{aac}-\beta_{aca}-\beta_{bbc}+\beta_{bcb})\\ +\langle \text{sin}\;\theta \;\text{cos}\;\theta \;\text{sin}\;\psi \rangle (\beta_{bab}-\beta_{bba}-\beta_{cac}+\beta_{cca})\\ +\langle \text{sin}\;\theta \;\text{cos}\;\theta \;\text{cos}\;\psi \rangle (-\beta_{aab}+\beta_{aba}-\beta_{cbc}+\beta_{ccb}) \end{array}\right\} \end{array}$$

(9) $$\begin{array}{l} \chi_{xyz}^{\left(2\right)}=\frac{1}{2}N_{S}\times \\ \left\{\begin{array}{l} \langle {\text{cos}}^{2}\;\theta \rangle (\beta_{abc}-\beta_{bac})\\ +\langle {\text{sin}}^{2}\;\theta \;{\text{sin}}^{2}\;\psi \rangle (\beta_{cab}-\beta_{acb})\\ +\langle {\text{sin}}^{2}\;\theta \;{\text{cos}}^{2}\;\psi \rangle (\beta_{bca}-\beta_{cba})\\ +\langle {\text{sin}}^{2}\;\theta \;\text{sin}\;\psi \;\text{cos}\;\psi \rangle (\beta_{aca}-\beta_{bcb}-\beta_{caa}+\beta_{cbb})\\ +\langle \text{sin}\;\theta \;\text{cos}\;\theta \;\text{sin}\;\psi \rangle (\beta_{abb}-\beta_{acc}-\beta_{bab}+\beta_{cac})\\ +\langle \text{sin}\;\theta \;\text{cos}\;\theta \;\text{cos}\;\psi \rangle (-\beta_{aba}+\beta_{baa}-\beta_{bcc}+\beta_{cbc}) \end{array}\right\} \end{array}$$

Equations 8 and 9 can be further simplified by eliminating the zero hyperpolarizability *β* elements using Equation 7 and the symmetry of the vibrational modes. Hence, a combination of Equations 3, 5, 6, 8, and 9 provides an expression of the experimental observable *I_SFG_* as a function of *β_ijk_* and molecular orientation (*θ*, *ψ*) at interfaces.

Therefore, chiral SFG spectra can provide information about molecular orientations and structures of chiral interfaces. For orientation information, if the microscopic molecular hyperpolarizability (*β*) tensor is known, the orientation (*θ*, *ψ*) can be obtained from the experimentally measured macroscopic second-order susceptibility elements, *χ_IJK_*^(2)^ (*I* ≠ *J* ≠ *K)*, as indicated by Equations 8 and 9. The microscopic molecular hyperpolarizability (*β*) tensor can be obtained either by linear IR [51] and Raman [52] measurements or by *ab initio* quantum chemistry calculations [53]. For structural information, vibrational peaks in the chiral SFG spectra can be assigned to particular vibrational modes of surface structures. This assignment is generally achieved by considering the selection rules, symmetries of vibrational modes, and normal modes analyses of the molecules under study. This approach for peak assignment can also be aided by *ab initio* quantum chemistry calculations. Such vibrational analyses and peak assignments can reveal molecular details of chiral interfaces.

### 2.4. The Origin of Chiral SFG Response

In the past decade, several research groups have developed theory to describe chiral SFG phenomena. Shen and coworkers applied first-order perturbation theory to describe the chiral signal detected from bulk chiral liquids and found that the anti-Stokes Raman tensor is responsible for the bulk chiral SFG signal [54]. Liu and coworkers also performed calculations to confirm that the chiral nonlinear susceptibility originates from the anti-symmetric component of the anti-Stokes Raman tensor [55]. The anti-symmetric part of the Raman tensor is usually very weak without electronic resonance. Thus, in order to detect this chiral SFG signal from bulk media, the visible beam is required to be in resonance with the electronic transition of the chiral molecules.

On the other hand, Simpson and coworkers also used perturbation theory with two-photon absorption to treat the molecular hyperpolarizability near and off resonance [56]. By analyzing the symmetry of vibrational modes, they determined the nonzero *β* tensor elements, from which they calculated the macroscopic second-order susceptibilit*y χ*^(2)^ of interfaces with *C_∞_* symmetry [50]. They further illustrated that achiral molecules, when arranged in macromolecular chiral architectures, can generate surface-specific chiral SFG signals [53,56–58]. This surface-specific chiral signal is comparable to conventional surface-specific achiral SFG without electronic resonance. Hence, the origin of chiral SFG signal generated at interfaces is different from the origin of chiral SFG signal generated in bulk solution. While the bulk chiral SFG signal is due to the asymmetric part of the Raman tensor associated with the “intrinsic chirality” of molecules, the surface-specific chiral SFG is due to chiral arrangements of chiral or even achiral molecular entities, whose microscopic hyperpolarizabilities are summed up to generate macroscopic chiral susceptibility elements at interfaces. Hence, when chiral SFG is employed to study chiral macromolecular structures, such as protein secondary structure, it can provide vibrational information about the macroscopic chiral structures that is surface-specific.

Furthermore, the origin of the chiral sensitivity of SFG is also different from that of linear spectroscopies, such as CD and ORD. For chiral SFG, the chiroptical response comes from the orthogonal elements of a third-rank susceptibility tensor, *χ_IJK_*^(2)^ (*I* ≠ *J* ≠ *K)*. Each of these third-rank tensor elements is described by three independent coordinates (*I*, *J*, *K*), which are sufficient to specify the symmetry of the interaction between optical fields and chiral molecular entities. For linear spectroscopy, however, neither the vector of electric dipole moments (*μ_k_*) nor the second-order tensor of Raman polarization (*α_ij_*) alone can reflect the chiral symmetry. Hence, in order to describe the chiroptical responses, the vector of electric dipole moments (*μ_ij_*) or the second-order tensor of Raman polarization (*α_ij_*) need to couple to magnetic dipoles, as in the case for CD or ORD. Such magnetic dipole coupling is generally weak. Hence, the sensitivity of CD or ORD is expected to be lower than chiral SFG.

## 3. Experimental Setup

Chiral SFG experiments are performed using a vibrational SFG spectrometer, of which there are two types: the scanning SFG spectrometer and the broad-bandwidth SFG spectrometer. Scanning spectrometers use picosecond (ps) narrow-bandwidth IR and visible beams [59,60], while broad-bandwidth systems use a picosecond (ps) narrow-bandwidth visible beam and a femtosecond (fs) broad-bandwidth IR beam [61–64]. Scanning SFG spectrometers scan the IR frequency stepwise to collect data point by point, and typically require 20–30 min to take a spectrum covering ~100 cm^−1^. In contrast, broad-bandwidth spectrometers use femtosecond IR pulses, which have a bandwidth of 200– 400 cm^−1^. Hence, SFG spectra can be acquired shot by shot, providing resolution in both time and frequency domains to probe kinetic processes. This allows broad bandwidth spectrometers to capture conformational changes of proteins at interfaces that happen on the time scale of minutes. This capacity is extremely useful to study kinetic processes of biomolecules at interfaces.

**Figure 4:**
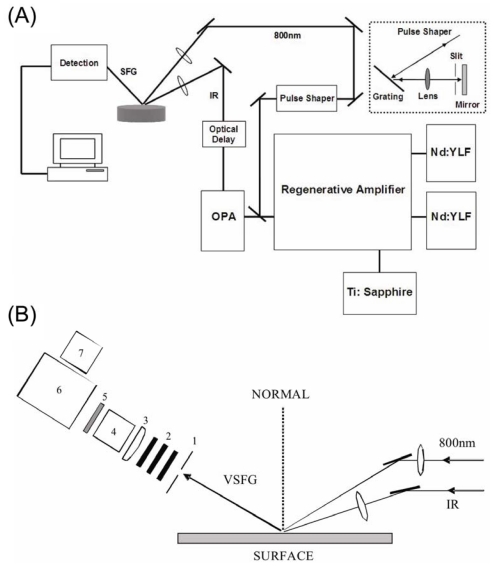
(**A**) Broad-bandwidth SFG spectrometer; (**B**) Detection system of the SFG spectrometer. 1: iris, 2: filters, 3: lens, 4: polarizer; 5: half-waveplate, 6: monochromator, and 7: CCD. Reprinted from ref [60], Ma *et al*. (2009). Copyright 2009 Society for Applied Spectroscopy.

Figure 4 shows a broad-bandwidth SFG spectrometer recently set up in our laboratory [65]. This spectrometer contains a 6-W regenerative amplifier seeded by a 120-fs 1.9-W Ti:sapphire oscillator (Mai Tai, Spectra-Physics) and pumped by two Nd:YLF pump lasers (16 W, Empower, Spectra-Physics). Half of the amplifier output (3 W) pumps an OPA (TOPAS, Spectra-Physics) to generate a 120-fs pulsed IR beam in the range of 3800–900 cm^−1^. The other half (3W) of the amplifier 800-nm output enters a pulse shaper to yield ~2 ps pulses to a narrow bandwidth of ~7 cm^−1^. The pulse shaper consists of a grating, a planoconvex cylindrical lens, and a homemade slit. The bandwidth of 800-nm beam can also be narrowed by the use of etalon as described by Lagutchev *et al.* [66]. The 800-nm and IR beams are focused below the sample surface to minimize photo-damage. They are tuned to overlap temporally and spatially at the interface. The reflected SFG signal is filtered, focused onto the slit of the monochromator (SP-2558, Princeton Instruments), and detected by a CCD (Spec-10:400BR/LN, Princeton Instruments) as shown in Figure 4B.

The SFG instrumentation has been rapidly advancing. High-power pulsed laser instruments have become user-friendly. Benderskii and coworkers have first implemented the heterodyne detection technique that enhances sensitivity and allows measurements of the absolute phase of the SFG optical field [67,68]. Several groups are currently building instruments for the two-dimensional SFG experiments. The Borguet and Zanni groups have reported results of using the 2D-SFG to probes local interactions and dynamics of molecules [69,70]. These advancements have broadened the horizon of applying SFG to gain dynamic and structural information to understand interfacial phenomena at high structural and time resolution. Further combinations of these advanced methods with chiral SFG spectroscopy hold promises to reveal molecular mechanisms of biological systems at interfaces that cannot be studied using conventional methods.

## 4. Experimental Observations and Biological Applications of Chiral SFG

### 4.1. Chiral SFG with Electronic Resonance

In 2000, Shen and coworkers detected chiral SFG signal from bulk chiral liquids using transmission optical geometry with electronic resonance enhancement [32]. They demonstrated, for the first time, the chiroptical response in an SFG process. They showed that chiral SFG spectral are useful for determining absolute configurations and conformations of chiral molecules [32,71–73]. Moreover, Ishibashi and coworkers probed porphyrin aggregates by double-resonance chiral SFG [74], and Busson and coworkers studied the anisotropy of a helicene bisquinone sample [75]. Both studies made use of electronic resonance to enhance the vibrational SFG signals. These chiral SFG signals, originating from “intrinsic chirality” of molecules, are due to the asymmetry of the Raman tensor and are usually weak. In order to detect these signals, the “interference” or “mix” methods are often applied, which use *pmp* (the polarization of the visible beam is in the “*m*” configuration, where “*m*” means 50% *p*-polarized and 50% *s*-polarized) rather than *psp* or *spp* polarizations settings [32].

### 4.2. Chiral SFG for Probing Biomolecules at Interfaces

**Figure 5:**
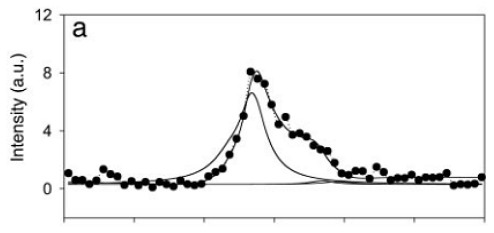

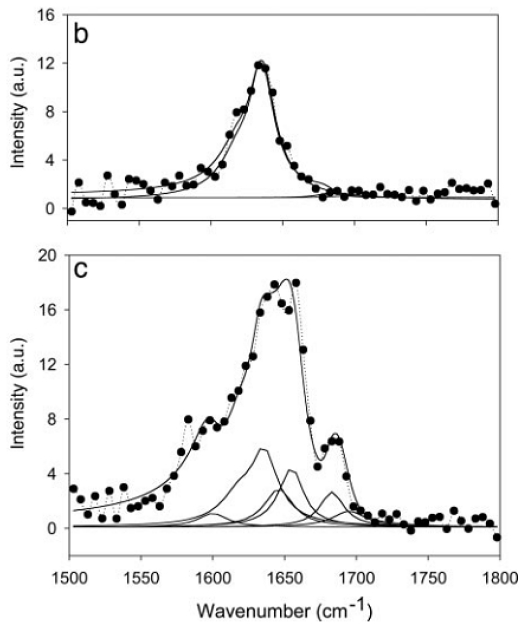
SFG spectra of tachyplesin I at the water/polystyrene interface obtained using the *psp* (**a**) and *spp* (**b**) polarization for chiral SFG experiments, and the *ssp* (**c**) polarization for achiral SFG experiments. Tachyplesin I was dissolved at a concentration of 0.1 mg/mL in phosphate buffered saline at pH 7.4. Reprinted from ref [33], Wang *et al*. (2005). Copyright 2005 the National Academy of Sciences.

Without electronic resonance enhancement, the chiral SFG signals have also been observed in various biological systems. Chen and coworkers detected chiral amide I signals using the *psp* and *spp* polarization configurations from a peptide, tachyplesin I, which forms an anti-parallel β-sheet structure on a polystyrene surface [33]. They made the first observation of the vibrational chiroptical signal from a protein using chiral SFG and identified three major peaks at 1633, 1685, and 1695 cm^−1^ in the amide I vibrational regions (Figure 5a and b). The amide I peaks at high frequency (>1680 cm^−1^) are characteristic of anti-parallel β-sheets. Although it was controversial whether this chiral SFG signal was due to surface chirality [71], the interpretation of surface chirality is supported by a theory developed by Simpson *et al*. [53,56–58]. According to the theory, the chiral SFG signals observed from proteins were attributed to the macroscopic chiral arrangement of the amide groups along the peptide backbone adopting anti-parallel β-sheet structures at interfaces. Chen’s pioneering work has demonstrated the potential of developing chiral SFG into a characterization method to probe protein structures at interfaces.

**Figure 6:**
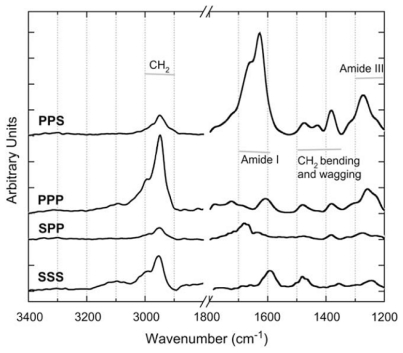
SFG spectra of collagen type I. The spectra were obtained using a scanning SFG spectrometer and the *pps* (chiral), *ppp* (achiral), *spp* (chiral), and *sss* (achiral) polarization settings. Reprinted from ref [71], Rocha-Mendoza *et al*. (2007). Copyright 2007 Elsevier.

Moreover, Knoesen and coworkers also applied chiral vibrational SFG to study the molecular origin of the second-order optical nonlinearity of collagen [76]. They obtained chiral and achiral SFG spectra of type I collagen in both the C-H stretch and amide I vibrational regions (Figure 6). They observed the chiral C-H stretch and amide I signals of type I collagen using the *spp* polarization configuration.

In addition, chiral SFG can also be used to determine orientations of proteins at interfaces. Using Simpson’s theoretical treatment, Chen and coworkers derived an analytical expression to describe the chiral amide I signals detected from anti-parallel β-sheet at interfaces, and successfully obtained its interfacial orientation at the polystyrene surface [38]. They analyzed both *ssp* and *psp* SFG spectra and expressed the effective second-order susceptibility, *χ_ssp_*^(2)^ and *χ_spp_*^(2)^, as functions of nonzero hyperpolarizability tensor elements, *β_ijk_*, and molecular orientations (*θ*, *ψ*), similar to Equations (8) and (9) presented above. They considered the *D_2_* symmetry of anti-parallel β-sheet and determined the non-zero derived *β_ijk_*, from which they derived the expression connecting *χ_ssp_*^(2)^ and *χ_spp_* ^(2)^ with nonzero *β_ijk_* and orientation (*θ*, *ψ*) for three amide I modes. Combining these results with the ATR-FTIR data and using the previously reported values for IR dipole and Raman polarizability, they successfully determined the orientation of the anti-parallel strands at the air/polystyrene surface.

In addition to proteins, it is worth mentioning that Geiger and coworkers also used chiral SFG to study DNA at interfaces by detecting the chiral C-H stretch. Their work suggests that the applications of chiral SFG to biological systems is not limited to proteins but can also be extended to other native or even synthetic chiral polymers [36,77].

The experimental results summarized in this section represent the current status of SFG applications in molecular chirality. The results demonstrate that chiral SFG is an effective method for detecting chirality at interfaces, which has inspired us to further develop chiral SFG as an analytical tool for *in situ* and real-time characterization of proteins at interfaces. In the following section, we will present our recent work on using chiral SFG for identifying secondary structures of proteins at interfaces [35] and for probing kinetics of conformational changes of amyloid proteins at membrane surfaces [37].

### 4.3. Chiral SFG for Characterization Protein Secondary Structures

We recently reported both chiral amide I and N-H stretch signals of peptide backbones from various secondary structures [35]. We obtained chiral (*psp* polarization) and achiral (*ssp* polarization) N-H stretch and amide I spectra of model peptides and proteins. The chiral SFG spectra of the model peptides and proteins have shown vibrational signatures unique to their secondary structures, supporting that chiral SFG can be used to distinguish protein secondary structures at interfaces. The model peptides and proteins that we studied include (1) human islet amyloid polypeptide (hIAPP), which forms parallel β-sheets at the air-water interface in the presence of negatively charged lipids [37,78–80]; (2) tachyplesin I, which has two intra-strand disulfide bonds stabilizing an anti-parallel β-sheet structure [81–83]; (3) bovine rhodopsin, which has a 7-α-helical transmembrane structure [84,85]; (4) pH-low insertion peptide (pHLIP), derived from helix 3 of bacteriorhodopsin, which adopts α-helical structures in amphiphilic environments [86,87]; (5) *de novo* designed LKα14, which forms α-helices at the air/water interface [88–90]; and (6) rat islet amyloid polypeptide (rIAPP) used as a control, which is largely disordered [91]. These proteins and peptides were studied at the plain air/water (phosphate buffer, 10 mM, pH = 7.4) interface, except for hIAPP and rhodopsin. For hIAPP, we added dipalmitoylphospho–glyc–erol (DPPG) to induce formation of parallel β-sheet structures. For rhodopsin, we made a monolayer of rhodopsin in the presence of detergent in a Langmuir trough by controlling the surface pressure as described [92,93].

**Figure 7:**
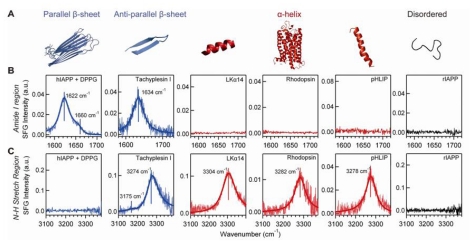
The chiral SFG spectra of the model peptides and proteins at interfaces obtained using the *psp* polarization setting. (**A**) Schematics of secondary structures of the hIAPP aggregate, tachyplesin I, LKα14, rhodopsin, pHLIP, and rIAPP. The chiral SFG spectra at the air-water interface in the (**B**) amide I and (**C**) N-H stretch regions. Adapted with permission from ref [35], Fu *et al*. (2011). Copyright 2011 The American Chemical Society.

**Figure 8:**
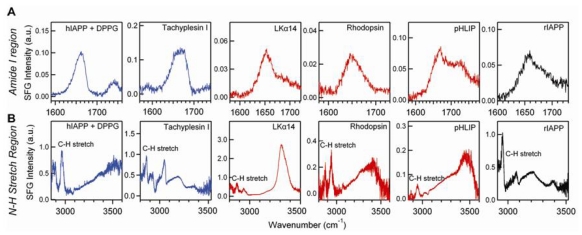
The achiral SFG spectra of the hIAPP aggregate, tachyplesin I, LKα14, rhodopsin, pHLIP, and rIAPP at the air/water interface obtained using the *ssp* polarization setting in the (**A**) amide I and (**B**) N-H stretch regions. Adapted with permission from ref [35], Fu *et al*. (2011). Copyright 2011 The American Chemical Society.

Figure 7 shows the chiral SFG spectra of the model peptides and proteins. The parallel β-sheet in hIAPP aggregates exhibits amide I peaks at 1622 and 1660 cm^−1^, but no chiral N-H signal. In contrast, α-helices display N-H signals at 3280 cm^−1^ (rhodopsin and pHLIP) and 3300 cm^−1^ (LKα14), but no amide I signal. Antiparallel β-sheets of tachyplesin I displays chiral N-H at 3274 cm^−1^ and 3175 cm^−1^, as well as chiral amide I signals at 1634 cm^−1^. Figure 8 shows the corresponding achiral SFG spectra taken using the *ssp* polarization setting. The achiral SFG spectra show both amide I and N-H stretch achiral signals at the interface regardless of their secondary structures. Compared to chiral SFG results, the peaks in achiral SFG spectra are generally broad and complex, and need spectral deconvolution to extract structural information. Taken together, chiral SFG shows an advantage over conventional (achiral) SFG by providing background-free and optically-clean signals for robust characterization of protein secondary structures at interfaces.

Figure 7 shows that the N-H stretches of peptide backbones can be used for characterizing protein structures at aqueous interfaces, which is unprecedented. Because the N-H stretch overlaps with the O-H stretch, the N-H stretch of proteins is often masked by water background in conventional vibrational spectra. However, the N-H stretch along chiral peptide backbones can be detected in chiral SFG spectra, but the O-H stretch of water, which lacks any chiral macroscopic structure, is muted in the chiral SFG spectra. Hence, the chiral N-H stretch of peptide backbones can be detected without interference from the water O-H stretch background. Moreover, specific hydrogen bonds between peptide amide (N-H) and carbonyl (C=O) moieties stabilize secondary structures, where the N-H stretch frequency is sensitive to the local hydrogen-bond environment. Hence, the chiral N-H frequency is expected to be characteristic of protein secondary structures, providing additional vibrational signatures to characterize protein secondary structures at interfaces. Nonetheless, the chiral N-H stretch for different secondary structures may overlap. Figure 7 shows similar peak positions for anti-parallel β-sheet tachyplesin I (3274 cm^−1^) and α-helical pHLIP (3278 cm^−1^). Hence, the chiral N-H stretch frequency by itself does not distinguish these two structures. However, β-sheet tachyplesin I shows a chiral amide I SFG signal, while α-helical pHLIP does not display any chiral amide I SFG signal. Thus, our results show that the chiral N-H stretch signal combined with the chiral amide I signal of peptide backbones can be potentially developed as robust optical probes for characterizing protein secondary structures at interfaces.

### 4.4. Chiral SFG for Monitoring Protein Folding at Interfaces

**Figure 9:**
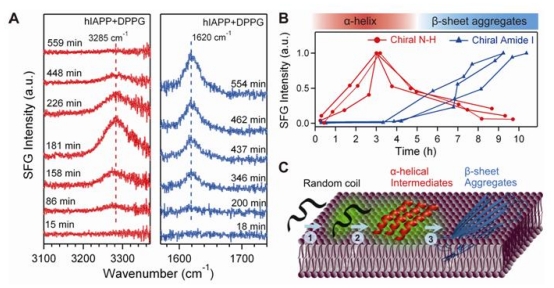
Aggregation of hIAPP. (**A**) The time-dependent chiral SFG spectra in the vibrational regions of N-H stretch (left) and amide I (right) after addition of DPPG; (**B**) The intensities of the N-H stretch and amide I signals as a function of time. Results of triplicate experiments are shown; (**C**) The aggregation model of hIAPP on a membrane surface as observed in the SFG experiments: adsorption as a random coil leads to formation of α-helical intermediates, which are then converted to β-sheet aggregates. Adapted with permission from ref [35], Fu *et al*. (2011). Copyright 2011 The American Chemical Society.

Here, we describe our recent application of chiral SFG to a biomedical problem. We used the SFG vibrational signatures of various secondary structures to study the kinetics of the misfolding of amyloid proteins at membrane surfaces. The misfolding is implicated in various amyloid diseases, including Parkinson’s, Alzheimer’s, and type II diabetes [94,95]. We focused on human islet amyloid polypeptide (hIAPP), which is associated with type II diabetes [96]. In the normal state, hIAPP is secreted by pancreatic β-cells in human body with random-coil structures [91]. In the disease state, hIAPP converts to aggregates in parallel β-sheet structures. Previous biochemical and biophysical studies showed that the aggregation of hIAPP into parallel β-sheet structures is catalyzed by its interactions with membrane surface [95–97]. We probed the aggregation process of hIAPP upon interactions with the negatively charged lipid, DPPG, at the air/water interface. We monitored the chiral N-H stretch and amide I SFG spectra over time. Upon addition of lipids (*t =* 0), the SFG intensity of the chiral N-H stretch gradually increased to its maximum in 3 hours and then disappeared by 10 h (Figure 9A). In contrast, the intensity of the chiral amide I signal appeared at approximately 4 hours after addition of lipid and increased to maximum by 10 h (Figure 9A). Each measurement was performed in triplicate and the N-H stretch and amide I intensities were plotted as a function of time in Figure 9B. The results show that the N-H stretch signal consistently disappeared prior to accumulation of the amide I signal. The transient N-H signal at 3285 cm^−1^ corresponds to an α-helical intermediate, while the amide I signal at 1620 cm^−1^ corresponds to a parallel β-sheet structure. The initial absence of amide I signal and N-H stretch signals reveal that hIAPP is likely unstructured upon adsorption onto the lipid surface. The gradual buildup of the amide I signal and a simultaneous decrease of the N-H chiral signal suggest the conversion of α-helices to parallel β-sheets. We conclude that hIAPP adsorbs at the lipid/aqueous interface as unstructured protein, which then folds into α-helical intermediates, and subsequently converts to parallel β-sheets on the time scale of 10 hours.

This application of chiral SFG to probe aggregation of amyloid proteins at interfaces has illustrated the potential of chiral SFG to address biomedical questions at the molecular level. It is expected that further application of chiral SFG for characterizing protein secondary structures at interfaces can solve problems that have biomedical relevance.

## 5. Advantages and Prospective of Chiral SFG

The chiral SFG studies summarized here show several advantages of chiral SFG for characterizing chirality of protein at interfaces. First, compared to traditional CD and ORD, which depend on the coupling with magnetic dipole, chiral SFG has higher sensitivity because the chiral SFG optical response originates from electric dipoles. Due to its surface-selectivity and relatively high sensitivity, chiral SFG spectroscopy can probe vibrational structures at interfaces using microgram quantities of samples, which can be useful for probing membrane proteins that are difficult to purify in large quantities. Second, chiral SFG provides vibrational signals in the visible region and utilizes femto-/pico-second laser pulses, which facilitate ultrafast dynamic studies. Third, it can probe protein orientations at interfaces, providing an extra handle to characterize protein conformations. Moreover, the chiral SFG signals in the N-H stretch and amide I regions are free of background from water; hence, H_2_O can be used as solvent. In addition, chiral SFG is muted for achiral solutes at interfaces, which can further reduce optical background. Finally, the spectroscopic signatures of secondary structures fall neatly into two regions: amide I (~1650 cm^−1^) and N-H stretch (~3300 cm^−1^), simplifying spectral deconvolution. These advantages make chiral SFG a promising surface-specific method to characterize protein structures at interfaces *in situ* and in real time.

Further development of chiral SFG as a surface characterization method for proteins is expected to have positive impacts in various research fields. It will aid molecular design of peptide-based biomaterials in material sciences for engineering functions, such as self-assembly, molecular recognition, and selective adhesion. By using a broad bandwidth spectrometer, the conformational changes of proteins at interfaces on the time scale of minutes can be monitored, enabling kinetic studies of protein folding on membrane surfaces. The chiral N-H signals provided by chiral SFG introduce a new approach to probe proton exchange in peptide backbones *in situ* and in real time, for revealing protein structures and dynamics at interfaces. Furthermore, the chiral SFG signals have structural selectivity and the capability of optical sectioning to yield 3D images. They are free of background from achiral solvents and solutes. Hence, chiral SFG can potentially provide ideal optical signals to overcome the current challenge in protein imaging—overwhelming background in cellular environments. Therefore, chiral SFG holds promises for wide application in material science, bioengineering, biomedical science, and beyond.

## 6. Conclusions

We have summarized the recent theoretical and experimental developments in chiral SFG spectroscopy in the past decades. These developments have established chiral SFG as a new approach to characterize chiral macromolecular structures at interfaces. Our review of chiral SFG theory is expected to provide a general understanding of spectral analysis and the molecular origins of chiral SFG signals. The introduction of the experimental setup of the SFG spectrometer and the references cited therein provide some general considerations for setting up spectrometers for chiral SFG measurements. The experimental results obtained using various molecular systems and the examples of applying chiral SFG to probe protein structures at interfaces have demonstrated the applicability of chiral SFG to solve problems in biomedical sciences. We hope that this review covering the experimental, theoretical, and application aspects of chiral SFG will provide an outlook for future developments of chiral SFG spectroscopy in probing chiral molecular structures and solving fundamental and engineering problems at the molecular level.
